# DNA Damage: A Main Determinant of Vascular Aging

**DOI:** 10.3390/ijms17050748

**Published:** 2016-05-18

**Authors:** Paula K. Bautista-Niño, Eliana Portilla-Fernandez, Douglas E. Vaughan, A. H. Jan Danser, Anton J. M. Roks

**Affiliations:** 1 Department of Internal Medicine, Division of Vascular Medicine and Pharmacology, Erasmus Medical Center, Rotterdam 3015 CN, The Netherlands; p.bautistanino@erasmusmc.nl (P.K.B.-N.); e.portillafernandez@erasmusmc.nl (E.P.-F.); a.danser@erasmusmc.nl (A.H.J.D.); 2 Department of Epidemiology, Erasmus Medical Center, Rotterdam 3015 CN, The Netherlands; 3 Department of Medicine & Feinberg Cardiovascular Research Institute, Northwestern University Feinberg School of Medicine, Chicago, IL 60611, USA; d-vaughan@northwestern.edu

**Keywords:** vascular, aging, endothelium, genomic instability, DNA damage, senescence, PAI-1, eNOS, phosphodiesterase, dietary restriction

## Abstract

Vascular aging plays a central role in health problems and mortality in older people. Apart from the impact of several classical cardiovascular risk factors on the vasculature, chronological aging remains the single most important determinant of cardiovascular problems. The causative mechanisms by which chronological aging mediates its impact, independently from classical risk factors, remain to be elucidated. In recent years evidence has accumulated that unrepaired DNA damage may play an important role. Observations in animal models and in humans indicate that under conditions during which DNA damage accumulates in an accelerated rate, functional decline of the vasculature takes place in a similar but more rapid or more exaggerated way than occurs in the absence of such conditions. Also epidemiological studies suggest a relationship between DNA maintenance and age-related cardiovascular disease. Accordingly, mouse models of defective DNA repair are means to study the mechanisms involved in biological aging of the vasculature. We here review the evidence of the role of DNA damage in vascular aging, and present mechanisms by which genomic instability interferes with regulation of the vascular tone. In addition, we present potential remedies against vascular aging induced by genomic instability. Central to this review is the role of diverse types of DNA damage (telomeric, non-telomeric and mitochondrial), of cellular changes (apoptosis, senescence, autophagy), mediators of senescence and cell growth (plasminogen activator inhibitor-1 (PAI-1), cyclin-dependent kinase inhibitors, senescence-associated secretory phenotype (SASP)/senescence-messaging secretome (SMS), insulin and insulin-like growth factor 1 (IGF-1) signaling), the adenosine monophosphate-activated protein kinase (AMPK)-mammalian target of rapamycin (mTOR)-nuclear factor kappa B (NFκB) axis, reactive oxygen species (ROS) *vs.* endothelial nitric oxide synthase (eNOS)-cyclic guanosine monophosphate (cGMP) signaling, phosphodiesterase (PDE) 1 and 5, transcription factor NF-E2-related factor-2 (Nrf2), and diet restriction.

## 1. Introduction

Cardiovascular diseases (CVD) are the leading cause of death worldwide, responsible for killing 17.3 million persons per year [1]. The onset of CVD is triggered by vascular endothelial alterations characterized by an impaired endothelium-dependent vasodilation, the overproduction of pro-inflammatory and prothrombotic molecules, and oxidative stress [2]. Age is the strongest independent predictor for CVD in risk scores in middle-aged persons, and an important determinant for cardiovascular health in the population aged 65 or older [3,4]. Aging is characterized by the complex interaction of cellular and molecular mechanisms that leads to a collection of functional problems. Focusing on the vasculature, such problems are closely associated with each other, and include worsened vasodilation, increased arterial stiffness and overt remodeling of the extracellular matrix, diffuse intimal thickening and a dysfunctional endothelium [4]. The mechanisms through which age actually contributes to cardiovascular risk remain the subject of speculation. From a classical perspective, modifiable risk factors promote and modulate molecular mechanisms that, as time progresses, culminate in an imbalance in the production *vs.* scavenging of ROS (*i.e.*, superoxide anions, hydrogen peroxide and hydroxyl radicals), increasing ROS levels, and, as a consequence, reducing the bioavailability of nitric oxide (NO) [5,6]. NO is crucial in the maintenance of vascular homeostasis, including in the regulation of vascular dilation, the modulation of cell growth and the prevention of thrombosis [7]. In the absence of a healthy endothelium, these factors gradually increase the pathologic phenotype of the vasculature up to the point that cardiovascular events occur.

**Figure 1:**
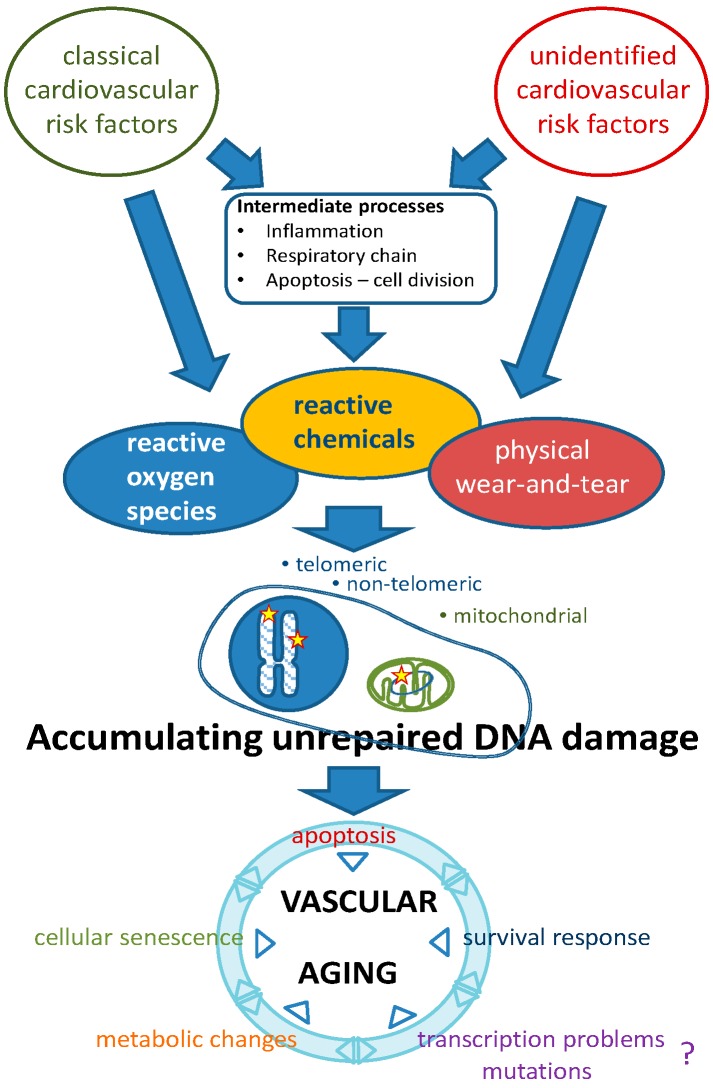
Etiology of vascular aging based on genomic instability as a causal factor. Classical and unidentified risk factors contribute to various types of DNA lesions. Unrepaired lesions accumulating during life lead to a growing set of pathophysiological changes that, either independently or in mutual interaction, lead to progressive vascular aging. The putative role of transcriptional problems or mutations herein needs to be established. The survival response may have beneficial (increased Nrf2-regulated antioxidants) as well as detrimental (decreased IGF-1 signaling, pro-inflammatory status) effects (see text and Ref. [8]).

While this paradigm explains vascular aging in view of classical risk factors as causative mechanisms, a recently proposed alternative view on vascular aging has emerged that presents new mechanistic alternatives for understanding the process of vascular aging [8]. In this novel paradigm, causal mechanisms for the process of aging itself, most notably genomic instability, including telomere attrition, drive the detrimental changes occurring increasingly with (biological) aging (Figure 1). The involvement of these causal factors of aging in general have been discussed elsewhere [9]. In the present review we summarize the evidence that supports the role of genomic instability in vascular aging. In addition, we present mechanisms through which genomic instability generates the functional changes that are typical for the aging vasculature.

## 2. Genomic Instability and Aging: A Short Outline of the Basic Principles

### 2.1. DNA Repair Systems

The maintenance of genomic integrity is critical for the prevention of aging of organisms. To safeguard genomic integrity, cells are equipped with several genomic maintenance systems that sense and repair DNA damage [10,11]. The sources of DNA damage are very diverse and range from intrinsic molecular reactions within DNA molecules such as hydrolysis, attacks by endogenous metabolic products, and ROS, to damage by exogenous physical and chemical entities such as chemotherapy and UVB light [12]. To account for the different types of DNA damage, cells are equipped with multiple DNA repair pathways. Each repair system is responsible for a specific subset of lesions, although partial overlap can occur depending on the type of DNA lesion that needs to be repaired. At least six DNA repair pathways can be listed in mammalian cells: (1) the direct reversal pathway, which executes the direct reversal of chemical modifications of nucleotides; (2) mismatch repair (MMR), which repairs base pair mismatches; (3) base excision repair (BER), repairing mainly oxidized and alkylation lesions in the nucleus and mitochondria, as well as single-strand breaks; (4) nucleotide excision repair (NER), to correct transcription-disturbing bulky adducts; (5) homologous recombination (HR); and (6) non-homologous end joining (NHEJ), which correct single- and double-strand breaks [10,13]. Telomere maintenance requires further specialized proteins [14]. Hypothetically, the classical cardiovascular risk factors initiate ROS-induced DNA damage and thus contribute to genomic instability-related vascular aging (Figure 1). Although some factors that lead to (vascular) genomic instability have been identified, the road to identification of all relevant contributors is still long (Figure 1) [8,15].

### 2.2. Aging: The Interplay between Genomic Damage, the Survival Response and Cellular Senescence

Unrepaired genomic damage enables the generation of harmful mutations that can be transferred to new cells during proliferation. This puts complex organisms at the potential risk of rapidly developing dysfunctional tissues or even tumors. As a protective measure, accumulating unrepaired DNA damage triggers a switch in biological pathways from a phenotype supporting growth to one favoring maintenance of the organism, a switch often referred to as the “survival response” [16]. However, the switch is believed to contribute to the typical changes that occur during aging, as demonstrated in humans and animals with defective DNA maintenance [16].

To avoid the harmful consequences of genomic instability, such as cancer, complex organisms have developed protective cellular mechanisms, namely apoptosis and cellular senescence. Whereas apoptosis embodies the loss of (dysfunctional) tissue due to programmed cell death, which might account for loss of organ function, cellular senescence has a more intricate relationship with aging tissues. Senescent cells undergo cell cycle arrest and thus can no longer replicate, although they remain metabolically active and often acquire a SASP, an immunogenic phenotype consisting of interleukins, pro-inflammatory cytokines, and growth factors [17]. It is believed that this results in an increased susceptibility to age-associated disease, including cancer and cardiovascular disease [17]. As a consequence of cellular senescence, the organisms age and become susceptible to age-associated diseases. Paradoxically, the accumulation of senescent cells with age, which is believed to result from an inefficient clearance by the immune system, might also help delay tissue dysfunction through cell loss. Recently, however, it was shown that removal of senescent cells expressing the cyclin-dependent kinase inhibitor p16^INK4A^ in genetically modified mice (INK-ATTAC mice) leads to a prolonged life and health span [18], supporting a fundamental role for cellular senescence in aging. The mechanisms through which removal of senescent cells leads to these effects remain to be elucidated.

## 3. Genomic Instability as a Causal Factor in Vascular Aging: Evidence in Humans

There is ample evidence that genomic instability is involved in vascular aging in humans. The following section highlights the observations that have accumulated until the present.

### 3.1. Cardiovascular Disease in Progeria Syndromes

The role of DNA damage in aging is further highlighted in human progeria syndromes. Human syndromes of progeria arise from mutations in genes involved in genomic maintenance in at least 75% of the known cases [19]. Progeria syndromes provide a unique opportunity to study aging, but it should be noted that they are not a complete phenocopy, e.g., progeria patients show phenotypes that are rare during normal aging, such as clavicular agenesis in Hutchinson-Gilford progeria syndrome or the intensified risk of cancer in Werner syndrome [20]. The relation of progeria to normal aging remains debatable.

Despite this continuing debate, it is intriguing to observe that several progeria syndromes manifest severe, juvenile cardiovascular disease. Werner syndrome (WS) is characterized by the premature onset of clinical signs of aging, such as cancer, osteoporosis and cardiovascular disease (diabetes mellitus type II and atherosclerosis) [21]. WS is caused by a WRN (Werner) gene mutation. WRN encodes a DNA helicase protein, *Escherichia coli* recQ-like helicase L2 (RECQL2), which is involved in DNA recombination, replication, repair and transcription, and also in telomere maintenance [22]. WS patients develop a considerable burden of atherosclerotic plaques in the coronary arteries and the aorta; calcification of the aortic valve is also frequently observed. Consequently, most WS patients die during middle age (average life expectancy is 46 years) due to myocardial infarction and stroke [21]. A related disease called Bloom syndrome, a consequence of mutation of the RecQ helicase gene *BLM*, features telangiectasias (dilated blood vessels in the skin), but the function of blood vessels has not been extensively investigated, although the occurrence of diabetes in these patients might be an important confounder in such investigations [23].

Hutchinson-Gilford progeria syndrome (HGPS), perhaps the best-known progeroid disorder, is characterized by hair loss, pain in the joints, wrinkled skin, and cardiovascular problems [24]. HGPS is caused, in most patients, by a point mutation in the lamin A gene (*LMNA*), which encodes the A-type nuclear lamins. The mutant lamin A, called progerin, remains fixed to the nuclear envelope causing various cellular changes, such as irregular nuclear shape and disorganization of heterochromatin, that lead to abnormal regulation of gene expression, therefore inducing premature aging. Death occurs around the age of 13 years mostly due to myocardial infarction or cerebrovascular events; however, in contrast to typical human aging or WS, atherosclerosis is very rare. Instead a major loss of vascular smooth muscle cells (VSMCs) in both big and small arteries is observed [25]. Interestingly, accumulation of prelamin A was observed in medial VSMCs and in atherosclerotic lesions from normally aged individuals. Moreover, prelamin A colocalized with β-galactosidase-positive VSMCs, *i.e.*, senescent VSMCs, and thus prelamin A was proposed as a marker of vascular aging in the general population [26].

Excision repair cross-complementation group 1 (ERCC1)-xeroderma pigmentosum (XP) F is a structure-specific protein complex serving as an endonuclease that participates in the repair of several types of DNA lesions, mainly bulky, helix-distorting lesions that are repaired by the NER pathway, but also double-strand breaks and interstrand cross-links [27,28,29]. Progeroid syndromes arising from ERCC1-XPF mutations, often unique cases as each of the mutations found until now has been encountered in individual patients, have been repeatedly reported as being characterized by hypertension [30]. This is further accompanied by frailty, loss of subcutaneous fat, liver dysfunction, vision and hearing loss, renal insufficiency, bone marrow degeneration, and kyphosis [31]. Although the hypertension observed in this syndrome might point at accelerated vascular aging, this still needs to be confirmed, certainly if one takes into consideration the presence of renal insufficiency in the patients suffering from this type of syndrome.

For other progeroid syndromes related to mutations in genomic DNA repair enzymes, data concerning vascular function are not available. It is uncertain whether this is an indication for the absence of vascular aging. Rather, more prominent problems in other organ systems or a focus on increased susceptibility to cancer might mask the presence of cardiovascular problems. In general, the patients are very frail, and cases are rare. Extensive cardiovascular characterization of such patients is, therefore, a very challenging task, and perhaps even not without risk for the patients themselves.

### 3.2. Indicators of a Role of Genomic Instability in the General Population

The role of genomic instability in disorders of the vasculature or the consequences thereof is a question that becomes increasingly important for the general population. If, indeed, this mechanism is central in age-related cardiovascular disease, there are major implications for prediction and detection and prevention. Research on the role of genomic instability in cardiovascular risk prediction opens a new window into expanding our understanding of the pathophysiology and causative risk factors in age-related diseases [8]. The use of emerging markers of DNA damage, identified in vascular and cardiac ischemic cells, has provided evidence for this role [32]. Part of the evidence comes from studies assessing the effect of ionizing radiation. An increased amount of circulating cell-free DNA and mitochondrial DNA (mtDNA) fragments has been observed in subjects exposed to low levels of ionizing radiation, suggesting the possible role of circulating DNA as a relevant biomarker of cellular damage [33]. In turn, it has been established that there is an association between radiation exposure and indicators of accelerated vascular aging, coronary artery disease and stroke in occupationally exposed groups. Andreassi *et al.* observed that long-term, low level radiation exposure is positively correlated to early atherosclerosis, as identified by increased subclinical cIMT (carotid intima media thickness), and to telomere shortening, an indicator for genomic instability [34]. This study also concluded that subjects with the Thr241Met polymorphism in the XRCC3 gene (gene coding for X-ray repair cross-complementing protein 3) have a greater susceptibility to radiation-induced vascular effects. Data of the Life Span study showed that people who had received an acute single dose of 1–2 Sv (sievert) had a significantly increased risk of mortality from myocardial infarction after 40 years of radiation exposure [35]. Other evidence is provided by observation of DNA damage markers in vascular tissue and circulating cells. Several groups observed elevated levels of oxidative DNA damage in human atherosclerotic plaques compared to non-atherosclerotic vessels or in circulating cells of persons with arterial disease [36,37]. Likewise, several proteins involved in DNA repair including DNA-dependent protein kinase (DNA-PK), poly (ADP-ribose) polymerase 1 (PARP-1), p53, and Apurinic/apyrimidinic endonuclease 1/redox factor 1 (APE-1/Ref1), were up-regulated in plaques of carotid endarterectomy specimens compared with non-atherosclerotic arteries [36]. On the other hand, genetic association studies have shown a significant association of single nucleotide polymorphisms (SNPs) in NER-related genes with age-related vascular phenotypes. In the population of the AortaGen Consortium, comprising 20,634 participants from nine cohort studies, Durik *et al.* identified an association of the SNP rs2029298 (*p*-value: 1.04 × 10^−4^) in the Damage-Specific DNA Binding Protein 2 (DDB2) gene with carotid-femoral pulse wave velocity, a measure of vascular stiffness [38]. In addition, suggestive associations were found for eight SNPs located within or near ERCC5, ERCC6, general transcription factor IIH (GTF2H) subunit 1 and 3 (GTF2H3, GTF2H1), and ERCC2 [38]. Verschuren *et al.* showed, in data from the GENDER (GENetic DEterminants of Restenosis) and PROSPER (Patient-centered Research Into Outcomes Stroke Patients Prefer and Effectiveness Research) studies comprising 6110 coronary artery disease (CAD) patients in total, that genetic variations in the NHEJ repair system are associated with risk for CAD [39]. In addition, several smaller studies have shown associations between polymorphisms in single DNA repair genes and risk of coronary artery disease, as reviewed elsewhere [40]. Interesting to note is also the finding that statins were found to improve DNA damage detection, which might be a mechanism leading to the improvement of atherosclerosis next to the reduction of lipids and oxidative stress [41,42].

### 3.3. Telomere Shortening

Human chromosomes are normally capped by telomeres that protect the end-segment of chromosomes between cell divisions. Since telomeres do not fully replicate during mitosis, they gradually become shorter as individuals age [43]. Defects in telomerase activity, abnormalities in DNA polymerase to synthesize terminal ends of the DNA, and the inhibition of the sheltering component telomeric repeat-binding factor 2 (TRF2) leads to an accelerated velocity of telomere shortening between cell divisions, which induces cellular senescence when the telomere reaches a critical length [43]. Telomere shortening promotes chromosome end fusion, chromosomal abnormalities and aneuploidy, suggesting that loss of chromosome end protection is correlated to genome instability [44]. Studies using knockout mouse models have established that the targeted deletion of 53BP1 and TRF2 genes is one of the main mechanisms involved in double-strand breaks and an increase of non-reciprocal translocations caused by telomere shortening [45]. In addition, RNA template of telomerase (TERC)^−/−^ and high mobility group box 1 (HMGB1)^−/−^ mice exhibit a reduced/null telomerase activity and telomere dysfunction, triggering aging-like cellular changes [46,47]. Population-based studies suggest that telomere shortening plays a role in the onset of vascular aging-related phenotypes. Individuals with a shorter mean telomere length exhibit a two-fold risk of abdominal aortic aneurysm compared to those with a higher telomere length (odds ratio = 2.30, *p* = 0.005) [48,49]. Moreover, an association between telomere shortening and the following CVD risk factors has been found: atherosclerosis, arterial stiffness (as measured by carotid-femoral pulse wave velocity), smoking, insulin resistance, type 1 and type 2 diabetes, obesity, hypertension and up-regulation of the renin–angiotensin–aldosterone system [50,51,52,53,54,55,56,57]. Likewise, an increased level of telomere shortening, via increased oxidative DNA damage, has been identified in circulating endothelial progenitor cells (EPC) in CAD patients with metabolic syndrome [58]. These observations suggest that oxidative stress-induced telomere shortening in EPC may accelerate vascular dysfunction, promoting the onset and progression of CAD due to a lack of vascular repair [58]. Despite the fact that the association of telomere shortening with aging and vascular-related disorders has been demonstrated, its potential use as a biomarker of age-related diseases remains unclear [59,60,61].

### 3.4. Cellular Senescence and Its Regulators

#### The Role of Senescent Cells and Plasminogen Activator Inhibitor-1 (PAI-1)-Related Signaling Pathways in Vascular Aging

**Figure 2:**
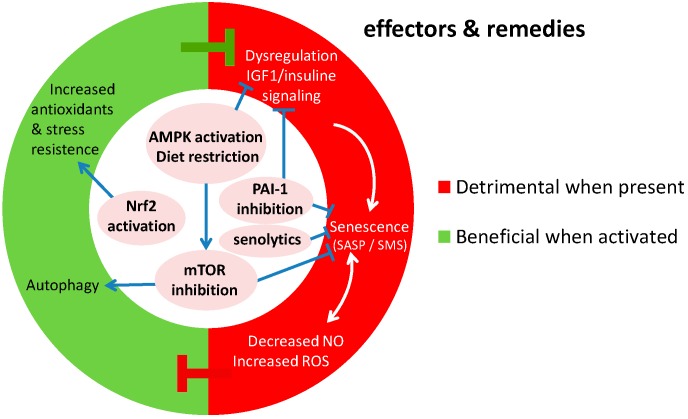
Molecular effectors of genomic instability that contribute to vascular aging, and the potential remedies (center of the chart) against that currently under development. Senescence, imbalanced NO *vs.* ROS production, inflammation and changes in insulin signaling are detrimental when present while autophagy, apoptosis and stress resistance have a beneficial contribution to vascular aging. IGF-1 putatively has a detrimental effect, although this needs further scrutiny (Ref. [8]). Pointed arrows indicate stimulatory processes, while blunted arrows indicate inhibitory processes.

As mentioned previously, genomic instability causes increased cellular senescence, which is an important candidate mechanism bridging the gap between DNA damage and vascular aging [8]. Senescent cells and tissues exhibit a distinctive pattern of protein expression, including increased plasminogen activator inhibitor-1 (PAI-1) as a part of the senescence-associated secretome (Figure 2) [62]. In addition to contributing to the molecular fingerprint of senescence, PAI-1 is essential and even sufficient for the induction of replicative senescence *in vitro* and is a critical downstream target of the tumor-suppressor p53 [63,64]. The contribution of PAI-1 to cellular senescence is broadly relevant in the organism as a whole, and age-dependent increases in plasma PAI-1 levels have been identified in wild-type mice as they age, in murine models of accelerated aging (*Klotho* and *BubR1^H/H^*), and in humans [18,65,66]. Partial and complete PAI-1 deficiency in *Klotho*-deficient animals (kl/kl) prevents telomere shortening and extends median survival up to 4.2-fold with substantial protection against aging-like phenotypes in various organs [67]. Furthermore, both genetic as well as pharmacological inhibition of PAI-1 protects against development of aortic arteriosclerosis in mice treated with long-term nitric oxide synthase inhibition [68].

**Figure 3:**
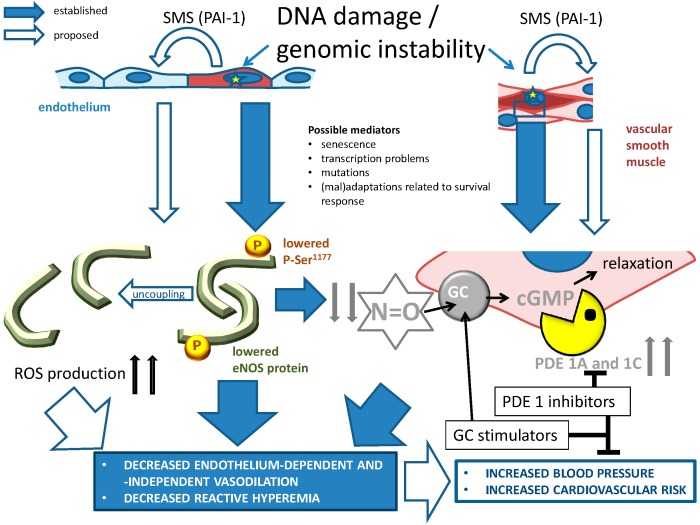
The role of genomic instability (indicated by yellow stars) on NO-cGMP signaling, and its consequences for age-related cardiovascular disease. Large, blue closed arrows indicate established relationships: genomic instability primarily leads to endothelial eNOS dysfunction in endothelial cells and to increased cGMP metabolism by PDE1A and 1C (Section 4.5.1). Large, blue open arrows refer to proposed mechanisms that were not fully explored: cellular senescence caused by unrepaired DNA could affect healthy cells through SASP/SMS, in which PAI-1 potentially plays a central role (Section 3.4.1 and Section 5.2.2.). The affected cells in turn might worsen vascular function through changes in eNOS-cGMP signaling. PDE1 subtype inhibitors and guanylyl cyclase (GC) stimulators are promising drugs to at least acutely improve vascular function. Their value for prevention of genomic instability and vascular aging needs to be assessed. PDE1A and 1C have a putative role in atherosclerosis, arteriosclerosis, reduced blood flow and hypertension (see Section 4.5.1 and Section 5.4). Their expression is strongly related to cellular senescence, and genetic variables of the PDE1A gene affect blood pressure and vascular hypertrophy (Section 4.5.1). Thus, both PDE1 subtypes appear to be central in vascular aging-related disease. Small, thick arrows pointing up or down indicate up- and down-regulation respectively. Blunt arrows indicate inhibition, pointed thine arrow indicate stimulation.

Metabolism also plays a fundamental role in the biology of (vascular) aging and iIGF1 are widely endorsed as critical contributors to senescence and aging in several experimental models (e.g., flies, worms, and mammals) [69]. In observational human studies, PAI-1 levels were increased in obesity and insulin resistance and independently predicted the future development of type 2 diabetes mellitus (T2DM) [70,71]. Potential anti-aging interventions have focused on caloric restriction and on drugs with metabolic effects, including metformin and resveratrol, all of which reduce PAI-1 expression [72,73,74,75]. Conversely, PAI-1 production is enhanced by insulin, free fatty acids, and glucose [76,77,78]. Taken together, these data suggest that PAI-1 and insulin exhibit a coordinated regulatory reciprocity, and in this context PAI-1 represents a high-yield translational target linking metabolism and biological aging, including aging of the vasculature (Figure 2 and Figure 3).

### 3.5. Cyclin-Dependent Kinase Inhibitor 2 (CDNK2) A and B

Further exploring the role of genomic instability-induced cellular senescence in vascular aging, gene variations in senescence regulators have been associated with age-related vascular disease in humans. Several studies have provided insight about the risk association of the 9p21 locus with aging-related cardiovascular diseases such as atherosclerosis and aortic aneurysm [79]. This chromosomal region codes for two cyclin-dependent kinase inhibitor genes, *CDKN2A*, comprising codes for p16^INK4A^ and p14^ARF^ (p19^ARF^ in mice), and *CDKN2B*, coding for p15^INK4B^. These CDKs are key molecules involved in the regulation of cellular replication, among others in vascular cells [80]. Genetic polymorphisms in this chromosomal region have indicated that 9p21 variation has a significant influence in the genetic expression of *CDKN2A* and *CDKN2B* in VSMCs, which could increase the susceptibility to CAD [81]. In addition, differential expression of *CDNK2A* and *CDNK2B* has been observed in senescent cells *in vitro* and in aging tissues of rodents and humans [82]. Thus, the measurement of the expression of these genes has led to their use as a potential biomarker of biological age [83]. Most of the studies have determined the role of *CDNK2A* and *CDNK2B* in aging by focusing on human tumors, concluding that the deletion and silencing of the *CDKN2A*–*CDKN2B* locus are among the most frequent genetic events found in human cancer cells [84]. Thus, *CDNK2A* and *B* play a central role in diseases of aging.

## 4. Genomic Instability as a Causal Factor in Vascular Aging: Evidence from Animal Models

### 4.1. Telomerase-Deficient (TERC^−/−^ and TERT^−/−^) Mice

Telomerase-deficient mouse models have been developed by knocking out TERC, TERC^−/−^ mice, or the telomerase reverse transcriptase (TERT^−/−^ mice). Homozygous TERT^−/−^ and TERC^−/−^ mice display short telomeres and a similar phenotype, but the TERC^−/−^ mice have been studied more comprehensively. The telomeres of the TERC^−/−^ mice shorten at a rate of ~5 kb in every subsequent generation (G). In conscious TERC^−/−^ mice, higher systolic blood pressures were observed in mice from G1 compared with wild-type mice, whereas in G3 mice, both systolic and diastolic blood pressures were increased compared with wild-type and G1 mice [85]. The differences in blood pressure between TERC^−/−^ and wild-type mice appear to be caused by an increased production of endothelin-1 in the TERC^−/−^ [85].

### 4.2. Mouse Models of Genomic Instability Associated with Human Progeria

Different mouse models of WS have been developed with either a complete knockout of the WRN protein, the transgenic expression of human WRN lacking helicase activity, or the in-frame deletion of the helicase domain. Depending on the model studied, the extent of the aging phenotype varies. The models lacking RecQ helicase activity show increased genomic instability and have increased visceral fat, high fasting triglycerides and cholesterol levels, insulin resistance and hyperglycemia [86]. Telomere shortening appears to be pivotal in the development of some of these metabolic changes [87], which are relevant analogues for human cardiovascular risk factors. In these models no vascular problems were reported, except, perhaps, decreased wound healing, which might implicate worsened angiogenesis.

A mouse model of HGPS expressing human progerin showed aberrations that were largely restricted to the vascular system. Recapitulating the vascular phenotype seen in patients with HGPS, these mice exhibited an increasing loss of VSMCs in the lamina media of large arteries [88]. Those changes were accompanied by a reduced vasodilator response to the NO donor sodium nitroprusside. Interestingly, the endothelium is initially undamaged, but with progression of the vessel damage, some loss in the endothelium is observed in 12-month-old mice [88].

Mice lacking proper function of the versatile DNA repair enzyme ERCC1 show many features of accelerated aging. *Ercc1*^−/−^ mice display a severe aging phenotype featuring frailty, osteoporosis, neurodegeneration, atrophic epidermis, sarcopenia, liver and kidney dysfunction and bone marrow degeneration [89]. *Ercc1^−/−^* mice only live four weeks, while mice with reduced ERCC1 function due to a combined exon 7 deletion allele and a null allele (*Ercc1^d/−^*, *Ercc1^−/Δ7^* or *Ercc1*^Δ/*−*^) live longer (up to 30 weeks). The *Ercc1*^Δ/*−*^ mice are healthy up to an age of eight weeks, when they start developing a gradual aging phenotype. In our previous study we found that *Ercc1*^Δ/*−*^ mice had an increased systolic blood pressure compared to wild-type mice [38]. They also display a decreased vasodilator response in their microvasculature [38]. Microvascular function was assessed by hind leg reactive hyperemia using a laser Doppler technique, which measures superficial resistance vessels and possibly represents both endothelium-dependent and endothelium-independent relaxations [90,91,92]. Those microvascular changes resemble the ones seen in aged rodents as well as in humans, and, strikingly, these changes in humans are at least partly independent of classical cardiovascular risk factors [90,93]. Another important characteristic of the human vascular aging phenotype that was recapitulated in the *Ercc1*^Δ/*−*^ mice includes greater stiffness, as measured by pressure-diameter relationships in the carotid arteries [38]. While aortic tissue from *Ercc1*^Δ/*−*^ contains increased amounts of senescent cells, the contribution of cellular senescence to vascular dysfunction remains uncertain [38].

Other genetic models add to the evidence, linking vascular senescence with vascular pathology and disease. Mice carrying the human XPD R722W mutation, so-called Xpd^TTD^ mice, show signs of accelerated vascular aging. In humans, the mutation in XPD causes trichothiodystrophy (TTD) which is characterized by postnatal growth failure, UV sensitivity, neurological degeneration, cachexia, osteoporosis and a shortened life span [94]. The Xpd^TTD^ mice show a similar phenotype, but the onset and severity of progeria is slower compared to *Ercc1*^Δ/*−*^ mice. We assessed vascular function in Xpd^TTD^ mice at 26 and 52 weeks and observed significantly reduced vasodilator responses to acetylcholine in aortic rings at 52 weeks compared to 26-week-old Xpd^TTD^ and wild-type mice [38].

### 4.3. Mitochondrial DNA Maintenance Defects

ApoE-deficient mice lacking protein kinase ATM (ataxia telangiectasia mutated), a protein pivotal in DNA damage detection, showed accelerated development of atherosclerosis [95]. This was attributed to increased mtDNA damage leading to malignant metabolic changes. Further exploring the involvement of DNA (mtDNA), polymerase gamma (POLG) performs DNA synthesis inside the mitochondria, and thus mutations in POLG cause mitochondrial disorders. A mouse model with an mtDNA mutator phenotype, conferred by a homozygous mutation in POLG, has been used to study the role of mitochondrial function and aging. In early adulthood the POLG mutant mice develop many features of premature aging such as weight loss, reduced subcutaneous fat, kyphosis, osteoporosis, cardiomyopathy and a reduced life span [96]. Oxidative stress and respiratory chain dysfunction due to the accumulation of mtDNA point mutations in protein-coding genes of the respiratory chain complexes are considered to contribute to the premature aging phenotype of the POLG mutator mice. Using a double POLG/ApoE low-density lipoprotein (LDL) receptor knock-out, it was shown that instability of mtDNA might contribute to atherosclerosis. POLG^−/−^/ApoE^−/−^ mice had increased atherosclerosis in the brachiocephalic artery and descending aorta as compared to POLG^+/+^/ApoE^−/−^ controls. The POLG^−/−^/ApoE^−/−^ mice also exhibited reduced body weight, reduced fat mass, hyperlipidemia and apoptosis of VSMCs [97]. Apart from vascular effects, POLG mutant mice develop cardiac hypertrophy and dilatation, impairment of systolic and diastolic function, and increased cardiac fibrosis within 13 months of age [98]. Overexpression of catalase in the mitochondria of these mice attenuated the malignant cardiac phenotype, providing evidence for the role of oxidative stress in the development of cardiomyopathy due to mtDNA instability.

### 4.4. BubR1 Knockout

The spindle assembly checkpoint protein BubR1 has an important role in the maintenance of genomic stability by ensuring the correct microtubule-kinetochore attachment and segregation of chromatids during mitosis [99].

Mice with reduced expression of BubR1 (10% of normal levels) develop progressive aneuploidy and exhibit a vascular aging phenotype characterized by reduced arterial elasticity, a reduced number of VSMCs, loss of endothelial-dependent relaxation, and increased production of superoxide anions. Apart from problems reminiscent of cardiovascular aging, BubR1 mice also show a variety of progeroid symptoms [100,101]. Also, naturally aged wild-type mice have decreased BubR1 expression in different tissues, suggesting that BubR1 may be a regulator in normal aging [18,27,100,101]. Clearance of p16^INK4A^-positive senescent cells with the INK-ATTAC strategy in BubR1 progeroid mice ameliorates several of the progeroid hallmarks. However, the cardiovascular problems are not rescued, which corresponds with the observation that these features are p16^INK4A^-independent in this model [102]. Importantly, vascular aging in ERCC1-defective mice appears to be associated with p53- and p21^Cip1^ (or p21^Waf1^)-related senescence [8,38], and this might further explain the ineffectiveness of removing p16-positive cells to improve cardiovascular function in BubR1 mice. Interestingly, it was recently shown that clearance of p16^INK4A^-positive senescent cells in non-progeroid mice increases the life span and reduces cardiac stress sensitivity [18,102]. This indicates that cellular senescence is indeed involved in deleterious cardiovascular phenotypes, involving both p16^INK4A^ as well as p53- and p21-related senescence in a differential way.

### 4.5. Vascular Functional/Pharmacological Changes Due to Genomic Instability

#### 4.5.1. NO-cGMP Signaling

NO is a key participant in many physiological pathways such as vasodilation, neurotransmission, and macrophage-mediated immunity. In the vascular endothelium, NO is produced from the substrate l-arginine by the enzyme eNOS (or nitric oxide synthase type III). The eNOS is activated by increased cytoplasmic Ca^2+^ levels, as induced, among others, by binding of vasodilatory (neuro)hormones to their G protein-coupled receptors [103]. Evidence provided up to date suggests that there is a reciprocal relationship between defective eNOS activity and genomic instability. During vascular aging, there is an increased production of ROS [104]. This ROS can be partly produced by eNOS, when the enzyme is in a so-called uncoupled state due to a reduced expression of the cofactor tetrahydrobiopterin (BH4), as has been shown in aging rats [105]. ROS coming from this and other sources, such as nicotinamide adenine dinucleotide phosphate (NADPH) oxidase or mitochondria, react with NO to form harmful free radicals, including peroxynitrite (ONOO^−^) and N_2_O_3_. The overproduction of ROS not only leads to a gradual reduction of NO bioavailability in the vasculature, but in addition can cause single-strand DNA breaks, 7,8-dihydro-8-oxoguanine and other oxidative lesions [106].

The aberrant eNOS function is closely associated with dysfunction as observed in aged and diseased blood vessels. In eNOS^−/−^ mice, systemic hypertension, altered vascular remodeling, dysfunctional angiogenesis and a prothrombotic phenotype have been observed [107,108,109,110,111]. In human atherosclerosis, eNOS mRNA expression was shown to be decreased in endothelial cells of advanced atherosclerotic plaques, which is accompanied by overt DNA damage [36]. In addition, eNOS uncoupling has been reported in patients with endothelial dysfunction as a consequence of diabetes, hypertension, hypercholesterolemia and smoking, linking the mechanism to classical risk factors [112].

Apart from a potential role of eNOS dysfunction in the production of DNA lesions, genomic instability itself causes dysfunction of NO signaling. Organ bath studies and molecular analyses in *Ercc1*^d/*−*^ mechanistically explained the decreased vasodilator responses [38]. These experiments showed that NO-mediated responses, eNOS expression, and eNOS activation through phosphorylation serine 1177 were decreased. Increased generation of ROS, a central mechanism in age-related decreased NO-dependent vasodilation, was partly responsible for the diminished vasodilation in *Ercc1*^d/*−*^ mice since anti-oxidants such as *N*-acetylcysteïne and BH4 improved vasodilation [38]. Therefore, faulty eNOS activation and genomic instability appear to form a vicious circle leading to progressive endothelial dysfunction and accelerated vascular aging (Figure 3).

Downstream of NO production, the ERCC1 functional mutation causes a pronounced defect in cGMP responsiveness. In the vascular function assessment of the Ercc1^d/*−*^ mice we found a strong reduction in the relaxations to the NO donor SNP compared to wild-type mice. The responses were completely dependent on soluble guanylyl cyclase (sGC) activity. No differences in sGC activity or protein levels were found between Ercc1^d/*−*^ and wild-type mice. When we measured the vasodilator responses of the aortic rings to SNP in the presence of vinpocetine, a phosphodiesterase (PDE) type 1 inhibitor, or Sildenafil, a PDE5 inhibitor, the dilator responses were increased. However, while in wild-type mice the greatest improvement was given by Sildenafil, in Ercc1^d/*−*^ mice it was given by vinpocetine [113]. This finding suggests that in Ercc1^d/*−*^ mice, a mouse model for accelerated aging due to genomic instability, PDE1 has a stronger role than PDE5 in regulating cGMP signaling and vasomotor function [113]; at least, it undergoes stronger regulation, because cellular senescence was shown to be strongly associated with increased expression of both PDE1A and C subtypes, and to a lesser extent with PDE5, in human cultured VSMCs. Supportive for a role in humans, PDE1A gene variations in human cohorts were associated with diastolic blood pressure and intima media thickness [113]. Together with previous observations showing associations of PDE1C with atherosclerosis, this places cGMP metabolism alongside these enzymes in the center of human vascular aging [114,115] (Figure 3).

#### 4.5.2. NF-E2-Related Factor-2 (Nrf2) and Antioxidant Pathways

NF-E2-related factor-2 (Nrf2) is a transcription factor activated in the vasculature to modulate the up-regulation of antioxidant genes, whose protein products are involved in the clearance of ROS and electrophilic molecules [116]. Consequently, Nrf2-dependent signaling pathways are activated as an adaptive mechanism in response to increased production of ROS, attenuating vascular oxidative stress and the damage caused by several stressors [117,118,119]. A dysfunction in Nrf2 action increases age-related cellular oxidative stress and cellular damage in aged vessels [120]. Nrf2 has a reduced function in senescence, whereas its silencing leads to premature senescence [121]. Remarkably, despite the fact that Nrf2 deletion leads to slower cell growth and shorter life span of individual cells in murine embryonic fibroblast cultures, it paradoxically induces immortalization in such cultures due to an early loss of p53 and p53-dependent gene expression [122]. This shows that Nrf2 loss-of-function has a dual pro-aging and oncogenic effect, centering the transcription factor in age-related disease. In addition, the effects of ROS associated with classical risk factors (smoking, hyperglycemia) in cardiomyocytes and mouse hearts are worsened after depletion of Nrf2, suggesting that Nrf2 protects against cardiac damage induced by mechanisms that can contribute to genomic instability [117,123,124,125]. *Xpg^−/−^* and *SIRT6^−/−^* mice, experimental models that exhibit progeroid phenotypes on the basis of genomic instability, have shown an accelerated cellular senescence, increased ROS levels and dysregulated redox metabolism. The Nrf2-regulated antioxidant pathways are up-regulated in the cerebellum and mesenchymal stem cells of these mice [126,127], which illustrates the physiological importance of Nrf2 as a line of defense against genomic instability caused by ROS. Importantly, Nrf2 is also involved in the regulation of production of NO *vs.* ROS through its role in eNOS uncoupling and activation [128]. This evidence suggests that Nrf2 provides a protective pathway during genomic instability, although the specific relation to vascular aging as caused by genomic instability has not been investigated yet.

## 5. Perspectives

### 5.1. Directions for Future Studies Establishing the Role of Genomic Instability

Although the evidence summarized above strongly indicates a major role of genomic instability in vascular aging, important questions remain to be solved. Firstly, there is the question as to whether the accelerated vascular aging features that are observed are due to local vascular processes, or whether they are the consequence of the general accelerated deterioration in mouse models of genomic instability. Tissue-specific inactivation of DNA repair enzymes is a means to explore this question. To this end, endothelial cell- and VSMC-specific Cre recombinase strains and mouse strains with Plox sites in DNA repair genes of interest are available, but still need to be combined. Furthermore, the mechanisms that are the interface between genomic instability and derailment of vascular signaling systems need to be resolved. Cellular senescence has been mentioned as an option, but apoptosis is also a candidate mechanism. The role of cellular senescence *vs.* vascular cell apoptosis in vascular aging remains an important question. Although previously discussed mouse studies provide compelling evidence, the role of cellular senescence *in vivo* in human aging remains unclear, mostly due to the absence of specific biomarkers that can provide information about the state of cells in tissues [129]. Restricting this to vascular aging, no conclusive evidence for a causal role of cellular senescence in vascular aging, let alone that induced by genomic instability, has been published yet. The aforementioned results in INK-ATTAC models on a wild-type mouse background, and in ERCC1-defective mice, are, however, highly indicative [18,38]. Models combining constructs to eliminate senescent cells in a background of vascular-specific genomic instability are putative tools to further establish this mechanism.

Another mechanism could be stem cell exhaustion, which requires comprehensive analysis of vascular cell progenitors in mice with increased genomic instability. In ERCC1-deficient mice, reduced hematopoietic progenitor cell reserves have been observed [130]. Since hematopoietic cells generate vascular progenitor cells [131], there is indeed a motive to explore this possibility.

Whether genomic instability in nuclear DNA outside of telomeres mediates vascular dysfunction through mutations or transcriptional dysfunction as caused by DNA lesions is a most important question that remains to be explored (Figure 1). Since DNA lesions have been repeatedly reported (see above), this is a very realistic option.

### 5.2. Towards New Interventions in Vascular Aging Caused by Genomic Instability

The awareness that genomic instability and cellular senescence arising thereof play a key role in general and in vascular aging opens new possibilities to prevent age-related cardiovascular disease. In particular, life-extending therapies that have been identified thus far are candidate interventions to decelerate vascular aging. In addition, interventions that prevent vascular genomic instability or readily improve NO-cGMP signaling are eligible for such purposes. We delineate the various options here.

#### 5.2.1. Mtor, Rapamycin and Autophagy

During aging, increasing dysfunction related to a progressive failure of maintenance and repair pathways takes place as aberrant macromolecules, dysfunctional organelles and DNA damage may accumulate in cells and tissues [132]. Therefore, cellular maintenance mechanisms are crucial to preserve normal cellular functions. Autophagy, one of the main cellular preservation processes, is involved in the degradation of long-lived proteins and dysfunctional organelles as well as in the maintenance of the cell in case of failure of macromolecule repair [133]. With age the rate of autophagy and protein degradation declines [134,135]. Importantly, genetic ablation of Atg7, an important mediator of autophagy, causes an accelerated appearance of vascular aging hallmarks in mice [136]. Autophagy-modifying drugs, such as rapamycin, inhibit the mammalian target of rapamycin complex 1 (mTORC1) and control the activation of autophagy-related signaling pathways. Rapamycin (also known as sirolimus) increases longevity and delays pathological lesions in mice [137]. Furthermore, the therapeutic use of rapamycin or related drugs prevents age-related diseases such as cancer and cardiovascular diseases in animal models [138,139]. In addition, the pleiotropic anti-atherosclerotic effects of rapamycin have allowed the implementation of rapamycin-based therapies to prevent or delay the pathogenesis of atherosclerosis [140]. Further, it has been reported that rapamycin improves endothelium-dependent vasodilation in old rodents [141]. A potential beneficial effect of mTOR inhibition on vascular aging independent from autophagy regulation was proposed [142]. This effect would be based on the regulation of a signaling network, consisting of mTOR, adenosine monophosphate-activated protein kinase (AMPK), and sirtuin (SIRT)-1. In this model, mTOR inhibition, in concert with SIRT-1 and AMPK activation, would counteract age-related vascular dysfunction thanks to modulation of the common transcription factors NFκB, FoxO and p53, that, when integrated, determine stress resistance, inflammation, ROS production, NO signaling, genomic instability and cellular senescence. Apart from this link to genomic instability and senescence, it has been found in a mouse model of Hutchinson-Gilford progeria that AMPK activation and mTOR inhibition occurs in conjunction with activation of autophagy [143]. Thus, models of genomic instability appear to implicate the proposed mTOR–AMPK signaling interaction, with a link to the regulation of autophagy (Figure 2). However, the effect on vascular aging as based on genomic instability remains to be explored.

Discouraging the use of mTOR inhibition is the fact that rapamycin significantly attenuates both endothelial function and the expression of eNOS in human endothelial cell lines *in vitro*, although it does not cause endothelial cell death [144]. Studies with mTOR-inhibiting drugs, among others applied on coronary stents in patients with advanced arterial aging, have reported deleterious effects of such drugs on various variables of endothelial (dys)function, although conflicting results are abundant [145,146,147,148,149]. It is unclear whether the conflicting results are dependent on the concentration of the mTOR inhibitor to which the endothelial cells are exposed, which presumably is very high in the case of drug-eluting stents. Although in cultured endothelial cells the increasing anti-inflammatory effect of increasing concentrations of mTOR inhibitors parallels the increasing cytostatic effect [150], this issue needs further inspection. We have also shown that rapamycin actually induces PAI-1 expression in cultured endothelial cells and *in vivo* in mice [151], so the net benefit of this drug in preventing senescence may be mixed at best.

In summary, mTOR inhibition, on the one hand, seems to be an attractive hypothetical option to reduce vascular aging in relation to genomic instability, but the idea should be approached cautiously.

#### 5.2.2. Senolytics and Inhibitors of Senescent Cell Signaling

Cellular senescence and the overproduction of SASP-associated proteins, also referred to as the senescence-messaging secretome (SMS), contributes to local and systemic dysfunction and disease. Therefore, the implementation of “senolytic” therapies has been approached as an intervention to specifically target senescent cells (Figure 2), eliminate them, and thus diminish the contribution of SASP and SMS [152]. The use of senolytic drugs including dasatinib and quercetin has been effective in eliminating senescent primary mouse embryonic fibroblasts and senescent human fat cell progenitors. *In vivo,* the combination of these drugs reduced senescent cells in normal aged, radiation-exposed mice, and in *Ercc1^−/Δ^* mice [152]. In addition, this study showed that periodic drug administration extended the health span in *Ercc1*^−/Δ^ mice and delayed age-related symptoms and pathology, osteoporosis, and loss of intervertebral disc proteoglycans. Despite the evidence suggesting that interventions that reduce the number of senescent cells could mitigate age-related tissue dysfunction, the burden of cell senescence biomarkers and SASP needs to be further studied and validated in humans. Therefore, the implementation of new therapies to reduce senescent cell number and SASP must be characterized.

Pioneer results from our group showed for the first time that modulation of the SMS can actually prevent the development of senescence in kl/kl mice, a mouse model of accelerated aging [67]. We observed that forced decrease of PAI-1 attenuated levels of the SMS factors insulin-like growth factor-binding protein 3 (IGFBP3) and interleukin-6 in plasma of kl/kl mice to levels seen in wild-type (WT) mice. In addition, telomere integrity was partially protected in numerous tissues. Furthermore, the nuclear accumulation of the senescence marker p16^INK4A^ was prevented. Similar observations were made in another aging-related model [68]. It is important to note that IGFBP3 is also strongly affected in DNA repair-defective progeroid models, as are other components of the IGF-1 growth factor signaling pathway, placing this pathway in the center of genomic instability-related (vascular) aging [8,153]. Moreover, this link raises the exciting possibility that PAI-1 might be involved in genomic instability-related vascular aging (Figure 2). As a still remote possibility, PAI-1 might act as part of the SMS from cells that become senescent due to unrepaired DNA damage, thus transmitting a harmful signal to cells in which the genomic integrity is still warranted (Figure 3). Application of genetic or pharmacological inhibition of PAI-1 in models of genomic instability is therefore an attractive approach to test this hypothesis.

### 5.3. Dietary Restriction

In search of treatment perspectives, it is of course important to consider more general anti-aging and longevity-increasing interventions. Apart from the previously discussed possibility to employ rapamycin against vascular aging, dietary restriction (DR) is perhaps the most important and well-known option. DR is a reduction of intake of food to the level that it results in low-normal levels of energy intake while avoiding malnutrition [154]. Claims of an effect of diet restriction on longevity date back as far as 3000 years. Studies that have taken place over many decades over the last century indeed confirm such an effect in various species, including yeast, worms, flies, spiders, rotifers, fish and rodents, demonstrating that DR is the most effective intervention to slow down aging and extend life expectancy [155,156,157,158,159,160,161].

DR is known also to protect against age-related cardiovascular disease. Two main mechanisms can be involved: (1) reduction of the intake of harmful food, such as carbohydrates and polysaturated fats [162,163]; or (2) slowing down of the aging process itself. It has been shown that chronic DR improves the aging-related rise of blood pressure and vascular wall remodeling, as shown in rodents [164,165]. This effect can be attributed to the improvement of vascular relaxation, a consequence of decreased ROS and increased NO bioavailability. In addition, DR has been reported to attenuate cardiovascular disease in nonhuman primates [74,166].

It is not clear what the main mechanisms of the anti-aging effect of DR are. However, the reduction of genomic instability is a possibility. In a previous review [8] we discussed that effects on oxidative stress-induced DNA and macromolecular damage are a putative mechanism. Reports have shown a possible effect of specific nutrient restriction and of caloric restriction on markers of DNA damage and DNA repair capacity, and a plethora of publications regarding the association between food consumption and telomere length is available [167,168,169,170]. This observation pleads for evaluation of the effects of DR on the general and vascular aspects of aging in models of genomic instability. Alternatively, effects on IGF-1/growth hormone (GH) signaling, SIRT-1 and nutrient-sensing pathways might be at play [8] (Figure 2). Since IGF-1/GH signaling is suppressed both after DR and in mouse models of genomic instability, this pathway apparently shares a common function in DR and the survival response in progeroid mice. Mouse models in which GH signaling is intentionally knocked out display increased longevity, and share features of the genetic program with genomic instability models [153]. Therefore, IGF-1/GH suppression is a point of convergence between DR, genomic instability and longevity. Whether this convergence takes place after genomic instability to improve survival, contributes to improved genomic integrity, as proposed above, or both remains to be elucidated. The effect of dietary restriction therefore needs to be explored in models of genomic instability, importantly those involving evaluation of vascular aging. The role of altered GH *vs.* IGF-1/insulin therein on vascular function needs special attention as these pathways appear to have opposite effects, as previously explained [8].

We here propose that the aforementioned relationship with mTOR and AMPK might also be important in DR effects (Figure 2). There is evidence that DR deactivates the mTOR-dependent signaling pathways, slowing aging and delaying aging-related diseases [171]. This suggests that DR and rapamycin can act together but have different effects on several pathways related to an increased longevity in young mice; therefore, the combination of both therapies could cause and exponential rise of lifespan in mice [172]. It would be interesting to investigate if such an interaction also exists for the attenuation of vascular aging.

### 5.4. PDE Inhibition

As mentioned before, there appears to be a pivotal role in vascular aging for PDE1 (Figure 3), and possibly also PDE5 [113]. At least, the inhibition of both PDE subtypes can acutely counteract diminished vasodilator responses caused by genomic instability [113]. Whether chronic treatment will also slow down vascular aging remains to be explored. An attractive aspect of PDE1 and 5 as drug targets is that there are several experimental and clinically approved drug candidates that might overcome the increased PDE activity. One is the selective PDE1 inhibitor IC86340, but unfortunately this drug appears not to be available anymore [173,174]. Other PDE1 inhibitors are under development [175]. Further, there is the possibility to inhibit PDE5, or both PDE1 and 5. Sildenafil is a PDE5 inhibitor which also blocks PDE1 at high doses [176]. Sildenafil was found to reduce both diastolic and systolic blood pressure in untreated hypertensive patients. However, due to Sildenafil’s short duration of action, research is focusing on new inhibitors such as tadalafil [177]. Vinpocetine is a PDE inhibitor with a preferential affinity for PDE1 over PDE5. Vinpocetine is an Food and Drug Adminstration (FDA)-approved nutriceutical and a registered drug in Eastern Europe, used to enhance cerebral bloodflow and improve memory [178]. PDE1 inhibitors were also developed for the treatment of cognitive impairment associated with schizophrenia [175]. Such treatment inhibits injury-induced hypertrophy in human and rodent vessels, and decreases atherosclerosis in ApoE knockout mice [179,180]. Therefore PDE1 inhibition is an attractive option for treating age-associated cardiovascular diseases. Until now, vinpocetine never found widespread application, for reasons that are unclear.

### 5.5. Reconsideration of Antioxidant Therapies

As explained above, ROS have been identified as a source of DNA damage, and therefore ROS scavenging is a potential treatment modality. Clinical studies applying ROS scavengers (antioxidants) have, however, not resulted in benefits for patients suffering from cardiovascular diseases [181]. Although this might be due to the fact that such interventions might require the onset of intervention early in life, there is also a shortcoming in that the drugs might not reach the right place at the right time or even hamper healthy cellular signaling that is performed by ROS [182]. A better targeted interaction of antioxidant enzymes and ROS might overcome the latter shortcomings of exogenously applied ROS scavengers.

Nrf2 has been proposed as a “master regulator” of cytoprotective mechanisms and it could be associated with increased longevity and attenuating age-related diseases in mice [183]. Therefore, Nrf2 gene regulation and the enhancement of the endogenous antioxidant capacity (Figure 2) could be an important therapeutic target to diminish the production of ROS, reducing DNA damage and their effects on vascular aging. Certainly, several drugs have been developed and tested to stimulate the bioavailability of NO through the regulation of the Nrf2/antioxidant response element (Nrf2/ARE). The combined action of NO and Nrf2/ARE signaling could improve vascular function and confer protection against vascular diseases [184]. On the other hand, several alternatives to increase Nrf2 have been currently explored, including calorie restriction, ozone therapy, hyperbaric oxygen and physical exercise [185].

## 6. Summary

There is ample evidence that genomic instability is involved in vascular aging. Nuclear DNA lesions, among which is telomere erosion, and mitochondrial DNA damage are strongly associated with several main features of vascular aging, such as diminished vasodilator capacity and increased vasoconstriction, increased blood pressure, increased vascular stiffness and atherosclerosis. Pivotal cellular biological changes involved in these pathological features comprise cellular senescence, apoptosis, autophagy, stem cell exhaustion and altered proliferative capacity of vascular cells. The role of gene mutation and of compromised transcription remains unknown (Figure 1). Potential mediating signaling pathways involved include components of the survival response (Figure 1), notably antioxidants under regulation of Nrf2 (beneficial), increased inflammatory status (detrimental) and decreased IGF-1/GH signaling (detrimental), as well as the interplay between mTOR, AMPK and NFκB, SIRT-1, and PAI-1, p53- and p21- and p16-related signaling. Proposed remedies against genomic instability-related vascular aging include PAI-1 inhibition, mTOR inhibition, DR, senolytics, PDE1 and 5 inhibitors and stimulators of Nrf2.

## Abbreviations

AMPK	adenosine monophosphate-activated protein kinase
APE-1/Ref1	Apurinic/apyrimidinic endonuclease 1/redox factor 1
ApoE	ApoE, Apolipoprotein E
ARE	antioxidant response element
ATM	ataxia telangiectasia mutated
BER	base excision repair
CAD	coronary artery disease
CDNK2	cyclin-dependent kinase inhibitor 2
cGMP	cyclic guanosine monophosphate
cIMT	carotid intima media thickness
CVD	cardiovascular diseases
DDB2	Damage-Specific DNA Binding Protein 2
DNA-PK	DNA-dependent protein kinase
DR	dietary restriction
eNOS	endothelial nitric oxide synthase
EPC	endothelial progenitor cells
Ercc1	excision repair cross-complementation group 1
GH	growth hormone
GTF2H	general transcription factor IIH
HGPS	Hutchinson-Gilford progeria syndrome
HMBG-1	high mobility group box 1
hMSC	human mesenchymal stem cells
HR	homologous recombination
IGF1	insulin-like growth factor 1
IGFBP3	insulin-like growth factor-binding protein 3
INK-ATTAC mice	genetically modified mice in which cells expressing the cyclin-dependent kinase inhibitor p16INK4A are being removed by apoptosis due to caspase 8 activation
LMNA	lamin A gene
MMR	mismatch repair
MtDNA	mitochondrial DNA
mTOR(C1)	mammalian target of rapamycin (complex 1)
NADPH	nicotinamide adenine dinucleotide phosphate
NER	nucleotide excision repair
NHEJ	non-homologous end joining
NfkB	nuclear factor kappa B
NO	nitric oxide
Nrf2	transcription factor NF-E2-related factor-2
PAI-1	plasminogen activator inhibitor-1
PARP-1	poly [ADP-ribose] polymerase 1
PDE	phosphodiesterase
POLG	polymerase gamma
RecQ	*Escherichia coli* recQ-like helicase
ROS	reactive oxygen species
SASP	senescence-associated secretory phenotype
(s)GC	(soluble) guanylyl cyclase
SIRT-1	sirtuin-1
SMS	senescence-messaging secretome
SNP	single nucleotide polymorphism
T2DM	type 2 diabetes mellitus
TERC	RNA template of telomerase
TERT	telomerase reverse transcriptase
TRF2	telomeric repeat-binding factor 2
TTD	trichothiodystrophy
VSMC	vascular smooth muscle cell
WRN	Werner gene
WS	Werner Syndrome
XP	xeroderma pigmentosum
XRCC3	gene coding for x-ray repair cross-complementing protein 3
